# Use of an exoscope for enhanced visualization of a Schwab grade 5 osteotomy to correct kyphotic deformity

**DOI:** 10.3171/2021.10.FOCVID21190

**Published:** 2022-01-01

**Authors:** Alma Rechav Ben-Natan, Nitin Agarwal, Saman Shabani, Jason Chung, Vivian Le, Dean Chou, Praveen V. Mummaneni

**Affiliations:** Department of Neurological Surgery, University of California, San Francisco, California

**Keywords:** exoscope, Schwab grade 5 osteotomy, intraoperative visualization, spine surgery

## Abstract

The development of the 3D exoscope has advanced intraoperative visualization by providing access to visual corridors that were previously difficult to obtain or maintain with traditional operating microscopes. Favorable ergonomics, maneuverability, and increased potential for instruction provide utility in a large range of procedures. Here, the authors demonstrate the exoscope system in a patient with progressive thoracolumbar junctional kyphosis with bony retropulsion of a T12–L1 fracture requiring a Schwab grade 5 osteotomy and fusion. The utilization of the exoscope provides visual access to the ventrolateral dura for the entire surgical team (surgeons, learners, and scrub nurse).

The video can be found here: https://stream.cadmore.media/r10.3171/2021.10.FOCVID21190

## Transcript

The following is a presentation regarding the use of the exoscope to visualize a Schwab grade 5 osteotomy.

### 0:26 Schwab Grade 5 Osteotomy Animation.

In a Schwab grade 5 osteotomy, the entire vertebral body is removed, followed by the removal of the superior and inferior discs. This allows for a 40° correction of kyphosis. A titanium cage may be used as a pivot point to correct the kyphosis.^1^

### 0:47 Clinical History.

This patient’s clinical history is the following: He is an octogenarian male. He presented with a few months of progressive back pain from T12–L1 fracture/kyphosis. He was initially treated at an outside hospital with a vertebroplasty. Subsequently, a CT and MRI demonstrate severe progressive thoracolumbar junctional kyphosis with bony retropulsion of the T12–L1 fracture, as well as some of the cement. The clinical and laboratory findings suggest he now has an infected vertebroplasty.

On physical exam the patient was severely limited by back pain and was unable to ambulate more than 30 feet.

Preoperative x-ray, MRI, and CT scan demonstrated a focal kyphotic deformity at T12–L1 with canal compromise. In addition, it is likely the vertebroplasty is infected based on contrast-enhanced imaging.

The patient was positioned prone with arms in the superman position on a Jackson table. Preoperative neuromonitoring baselines were obtained, and the mean arterial pressure was maintained throughout surgery at 90 to perfuse the spinal cord.

### 1:57 En Bloc Laminectomy.

Following exposure, pedicle screws were placed above and below the kyphotic deformity utilizing stereotactic navigation. Subsequently, an en bloc laminectomy was performed at T12–L1.

Additional temporary pedicle screws were placed at the fracture level. This was done to temporarily assist with screw rod kyphosis correction.

Further decompression of the spinal canal was done using a transpedicular approach at T12.

An osteotome was utilized to perform a complete facetectomy bilaterally at T11–L2 in order to fully decompress the exiting nerve roots and correct the kyphosis.

### 3:00 Schwab Grade 5 Osteotomy.

A combination of interbody shavers and pituitary rongeurs were used to perform the Schwab grade 5 osteotomy, also known as a vertebral column resection or a posterior-based corpectomy.

In order to achieve better visual access to the areas ventral to the thecal sac, the exoscope was utilized.^2–4^ This allowed for efficient removal of the bony elements and vertebroplasty cement ventral to the thecal sac. The surgeon, the assistant surgeon, and the scrub nurse could all see what was happening during the operation by watching the operation on the exoscope.^5–8^

The corpectomy at T12 was completed followed by the removal of the adjacent discs at T11–12 and T12–L1 and also removal of migrated pieces of vertebroplasty cement into the ventral thecal sac area.

An L-shaped tamp was then used to ensure no cement or bone were adherent to the ventral surface of the thecal sac.

During a Schwab grade 5 osteotomy visualization of the ventral surface of the dura is typically a limiting factor in both speed and safety. The exoscope allows access to visual corridors of the ventral dura that were previously difficult for the surgeon, the assistant surgeon, and the scrub nurse to all see simultaneously.^9^

### 4:44 Cage and Rods Placement.

An expandable cage trial was used to size the appropriate size expandable cage. Next, the titanium rods were contoured, placed, and sequentially reduced. An expandable cage was placed into the corpectomy site. Subsequently, screw on rod compression was done to reduce kyphosis using the cage as a ventral pivot point.

Appropriate placement of the cage was verified with fluoroscopy. The second rod and a cross-link was placed. Extracompartmental bone graft was added around the cage in the corpectomy site. The ventral surface of the thecal sac was assessed with a Woodson to ensure that there was adequate decompression.

### 6:46 Postoperative Imaging.

These final x-rays and CT demonstrate the postoperative correction and the final construct spanning from T10 to L3, along with a T12 Schwab grade 5 osteotomy, with the osteotomy site filled by an expandable cage.

## Disclosures

Dr. Agarwal reported “other” from Thieme Medical Publishers and Springer International Publishing, outside the submitted work. Dr. Chou reported personal fees from Globus and Orthofix, outside the submitted work. Dr. Mummaneni reported personal fees from DePuy Synthes, Stryker, and Globus; grants from ISSG, NREF, AO Spine, and NIH; and “other” from Spinicity/ISD, outside the submitted work.

## Author Contributions

Primary surgeon: Mummaneni. Assistant surgeon: Shabani, Agarwal, Chou. Editing and drafting the video and abstract: all authors. Critically revising the work: Shabani, Agarwal, Chou, Mummaneni. Reviewed submitted version of the work: Shabani, Rechav Ben-Natan, Agarwal, Chung, Chou, Mummaneni. Approved the final version of the work on behalf of all authors: Shabani. Supervision: Mummaneni.
